# MicroRNA-503 suppresses cell proliferation and invasion in osteosarcoma via targeting insulin-like growth factor 1 receptor

**DOI:** 10.3892/etm.2021.10845

**Published:** 2021-10-06

**Authors:** Zili Wang, Chenhuang Zheng, Kunqi Jiang, Jinshen He, Xu Cao, Song Wu

Exp Ther Med 14:1547–1553, 2017; DOI: 10.3892/etm.2017.4648

Following the publication of this paper, it was drawn to the Editors’ attention by a concerned reader that the Transwell cell migration assay data shown in Fig. 2C were strikingly similar to data appearing in different form in other articles by different authors. Owing to the fact that the contentious data in the above article had already been published elsewhere, or were already under consideration for publication, prior to its submission to *Experimental and Therapeutic Medicine*, the Editor has decided that this paper should be retracted from the Journal. The authors were asked for an explanation to account for these concerns, but the Editorial Office did not receive any reply. The Editor apologizes to the readership for any inconvenience caused.

